# Annotations

**Published:** 1894-08-11

**Authors:** 


### Annotations.

### Medical Ambitions.

Every intelligent doctor is pleased when some leader of his profession gives utterance to the deeper, wider, and more humane purposes which animate us and our art as compared with those which animated the art and purposes of our predecessors. Dr. Long Fox, who was elected President of the British Medical Association the other day, served his profession and the public well in the subject he chose for his inaugural address. He is aware that doctors are somewhat at a discount in the great councils of the nation. But we shall not be at a discount long, Dr. Fox holds; and that because we have deep and important truth in our professional arcana of a kind which is quite indispensable to the well-being of modern peoples and States. The day is fast coming when all educated persons will come to us with reasonable docility to learn what we have to teach them. They will not much longer try to hide their own ignorance of elementary science by the affectation of superior general knowledge. The State, says Dr. Fox, needs us for its advisers. It is not yet fully aware of its own needs in this direction. But it is our duty to teach it, that is to teach the public, and to persist in teaching until the whole State shall come to know its own physiological needs. There Dr. Long Fox struck a true, courageous, and patriotic note. We as a profession can do great things for our age and country if our age and country will but accept us as teachers and guides. Dr. Fox holds, as we hold, that though the ignorant and the half-cultured and the vain may reject us for a time, and may even try to inflict their patronage upon us, we must be neither discouraged nor disgusted; but with the sobriety and patience of true science we must continue to teach and persuade until all the intelligence of the country is enlightened and on our side. Our great ambition, says Dr. Fox, is the formation of a pure commonwealth, a commonwealth, that is, of healthy, intelligent, and thoroughly capable men and women. We could hardly have a nobler ambition; and we may well ask the world to try to understand our aims, and not to oppose, but to help us with all its energy to realise them as speedily as may be possible. If it be true that no nation ever has been great or ever can be great without the endowments of strong intellect and stern virtue, it is equally and almost, perhaps, more true, that no nation ever has been or ever can be great without a strong physique and healthy staying power.

### Influenza, a Theory of Cause and Cure.

Sir T. Grainger-Stewart, physician to the Queen for Scotland, gave the address in medicine to the members of the British Medical Association at Bristol last week, and took occasion to discuss fully and elaborately the etiology, prevention, and cure of influenza. Concerning the cause, Professor Grainger-Stewart is quite satisfied in his own mind that Pfeiffer's bacillus is the author of the mischief. Unfortunately, although this bacillus is found in abundance in the mucous secretions of the victims of influenza, it does not produce characteristic effects upon animals that are inoculated with it. This is peculiar, and suggests the propriety of a little scepticism. Most human pathogenic bacilli do produce their characteristic effects upon other animals; and the fact that Pfeiffer's bacillus fails here, raises a question which is not easily answered. For cure, Sir Thomas Grainger-Stewart is not quite satisfied with the "expectant" and "treat-symptoms" line of things. He desires to see something more definite done, as we all do. If another epidemic of influenza should arise, he intends to try some intra-tracheal injections of antiseptic substances, such as he has already found so serviceable in bronchiectasis and certain other diseases of the respiratory tract. Unfortunately, this is only a purpose and a good intention as yet, Professor Stewart not having apparently thought of such a method of treatment until the recent epidemic had passed away. For prevention, the Scotch Professor has some hope in inoculations. Pfeiffer, in this connection, inclines to the idea that one or other of the derivatives of the influenza bacillus, when isolated, may prove of some use as immunifying agents. But, as Sir Thomas Grainger-Stewart points out, an attack of influenza itself confers no immunity, and it does not therefore seem *a priori* probable that any mere derivative of the influenza bacillus will be more protective than millions of the bacilli, plus their derivatives, have proved to be. The subject is deeply interesting, and we could almost sigh for another slight epidemic in order that our newer theories might be properly put to the proof.

### The Babies of Battersea.

It is curious and interesting to "dig out," as it were, the idiosyncracies of different medical officers of health from the published pages of their annual reports. One medical officer of health, for example, will have a passion for "middens." Never a midden of the most inoffensive character in the remotest back-yard, but, in sporting phraseology, he will "nose" it out. Another is strong on water supply; a third goes for overcrowding, and so on. Dr. Kempster, the M.O.H for Battersea, cares little for these commonplace delights of the average M.O.H. Dr. Kempster puts his trust in babies. It is his special delight that his fine parish of Battersea is an excellent baby-thriving ground. Battersea has more babies in proportion to its population than any other parish in London. Whilst London generally could only show 31 new babies for every thousand of the population in 1893, Battersea showed 32·6. At the other, or funeral end of the scale, Battersea has an equally good eminence. All London killed 21·3 per thousand of its population in 1893, but Battersea put to death no more than 17·4 per thousand. Now there is some cause for these satisfactory facts. Is it that the air of Battersea is unusually good, or that the doctors are especially clever, or that the M.O.H. is a man of unusual capacity? We cannot tell. But we venture to offer Dr. Kempster our sincere congratulations upon the excellent health of his vast parish, and especially upon the marked prosperity of its babies.

